# Inflammasome Activation in Bovine Peripheral Blood-Derived Macrophages Is Associated with Actin Rearrangement

**DOI:** 10.3390/ani10040655

**Published:** 2020-04-10

**Authors:** Amin Tahoun, Kirsty Jensen, Hanem El-Sharkawy, David Gally, Amira M. Rizk, Jamaan Ajarem, Ahmed Allam, Ayman M. Mahmoud

**Affiliations:** 1 Division of Immunity and Infection, The Roslin Institute and R(D)SVS, The University of Edinburgh, Easter Bush, Midlothian EH25 9RG, UK; kirsty.jensen@roslin.ed.ac.uk (K.J.); david.gally@roslin.ed.ac.uk (D.G.); 2 Department of Animal Medicine, Faculty of Veterinary Medicine, Kafrelsheikh University, Kafrelsheikh 33511, Egypt; 3 Department of Poultry and Rabbit Diseases, Faculty of Veterinary Medicine, Kafrelsheikh University, Kafrelsheikh 33511, Egypt; hanem_amin@yahoo.com; 4 Department of Bacteriology, Mycology and Immunology, Faculty of Veterinary Medicine, Benha University, Benha 13511, Egypt; dr_az80@yahoo.com; 5 Zoology Department, College of Science, King Saud University, Riyadh 11451, Saudi Arabia; jajarem@ksu.edu.sa; 6 Zoology Department, Faculty of Science, Beni-Suef University, Beni-Suef 62514, Egypt; allam1081981@yahoo.com

**Keywords:** actin rearrangement, inflammasomes, macrophages, inflammation, flagellated bacteria

## Abstract

**Simple Summary:**

In the early stage of infection, the innate immune system produces a rapid inflammatory response that blocks the growth and spread of the infectious agent. In this study, we explored the role of the actin cellular cytoskeleton in the inflammatory response due to stimulation of the bovine macrophages with *Salmonella typhimurium* flagellin. We found that actin was rearranged to form filopodia, which in the early stage of inflammation are important for macrophage motility. As inflammation progressed, actin polymerized at the same site as inflammasome complexes formed. Ultimately the macrophage died, which will attract more inflammatory cells to the infection site to help block the infection.

**Abstract:**

Inflammation is critical for infection control and acts as an arsenal defense mechanism against invading microbes through activation of the host immune system. It works via its inflammasome components to sense the dangerous invading microorganism and send messages to the immune system to destroy them. To date, the function of bovine macrophage inflammasome and its relationship with actin has not been identified. This study aimed to investigate the activation of bovine inflammasome by phase one flagellin from *Salmonella typhimurium* and its interaction with actin. Bovine monocyte-derived macrophages were prepared and challenged with *S. typhimurium* SL1344 phase one flagellin. The results demonstrated the relationship between the flagellin-based activation of inflammasome and actin rearrangement. The flagellin-based activation of inflammasome promoted the activation and co-localization of F-actin and the inflammasome complex. Actin was remodeled to different degrees according to the stage of inflammasome activation. The actin redistribution varied from polymerization to filopodia, while at the stage of pyroptotic cell death, actin was broken down and interacted with activated inflammasome complexes. In conclusion, flagellin-dependent inflammasome activation and actin localization to the inflammasome at the stage of pyroptotic cell death may be of importance for appropriate immune responses, pending further studies to explore the exact cross-linking between the inflammasome complex and actin.

## 1. Introduction

Macrophages have complex functions in the immune system, playing an essential role in both innate and adaptive immunity [1,2]. Their role in innate immunity as phagocytic cells is a very crucial part of the host defense system [3]. Macrophages have the capacity to fight against different kinds of infections and to mount specific immunological responses [4,5]. Macrophages also play an important role in the adaptive immunity via antigen presentation and activation of T and B lymphocytes [3,6,7]. The macrophages’ recognition of different pathogens is conferred by multiple specialized cellular receptors that induce cellular signals to activate the immune system. These receptors include the pattern recognition receptors (PRR) of which the Nod-like receptors (NLRs) and Toll-like receptors (TLRs) are important to sense the pathogen associated molecular patterns (PAMP) of various micro-organisms [4]. One of the most important weapons of the macrophage arsenal is the activation of TLRs and inflammasomes that activate the host innate responses [8]. The inflammasome activation induces inflammation that helps in limiting the infection during the early stages [9]. Inflammasomes are groups of multi-protein complexes that are present in the cytosol [10,11]. The inflammasome complex comprises members of the NLR (nucleotide-binding domain leucine-rich repeat) family, which recognizes the PAMP and works as scaffolding protein for the activation of caspase-1. This complex also contains the apoptosis-associated speck-like protein containing a CARD (ASC) that connects caspase-1 to NLR [12,13]. The main part of inflammasome complex is caspase-1 which is an apoptotic cell death-inducing enzyme that cleaves and activates interleukin-1β (IL-1β) and IL-18 [11,14,15,16,17,18]. The active IL-1β is then released and induces inflammation and apoptotic like cell death known as pyroptosis [10,19,20].

There are many distinct inflammasomes classified according to their NLR protein [20,21]. Each member of the NLR superfamily has specificity for particular microbial products [10,20]. For example, NLR proteins of the NLRP1 family activate their inflammasome in response to anthrax lethal toxin (LT) [22]. The NLR protein nucleotide-binding domain, leucine-rich-repeat-containing family, pyrin domain-containing 3 (NLRP3) has been proposed to sense a wide range of stimuli including bacterial RNA, viral DNA, and uric acid crystals [10,20]. The canonical inflammasome protein NLRP3 recognizes the bacterial lipopolysaccharide (LPS) [21] and the NLRC4 inflammasome specifically recognizes bacterial flagellum in the cytoplasm and in response activates caspase-1 [23,24]. The non-canonical inflammasome activation depends on caspase 4/5 in humans or caspase-11 in mice and has been described to be pivotally involved in immune homeostasis [25]. Recently, Vrentas et al. [26] have characterized the NLRP1 inflammasome in primary bovine macrophages in response to anthrax LT and showed that its activation requires N-terminal cleavage. Inflammasome activation has also been shown to restrain the proliferation of *Neospora caninum* in bovine macrophages [27].

The macrophage is one of the non-muscle cells that possess actin stress fiber bundles [28]. Importantly, several lines of evidence pointed to actin cables as the major players of cell contraction with a comparable ability to actinomyosin of muscle cells [29]. Actin cables are composed of bundles of approximately 10–30 actin filaments [29]. These bundles are held together by the actin-crosslinking protein-actinin [30,31]. The mechanisms regulating the bovine inflammasome activation and its interaction with actin have not been fully elucidated. Herein, this investigation aimed to study the bovine inflammasome activation by phase one flagellin from *Salmonella typhimurium* and its interaction with actin. We described the features of bovine macrophages from bovine peripheral blood and the inflammasome activation with actin redistribution which might have a significant role in activation of the host immune response

## 2. Materials and Methods 

### 2.1. Preparation of Bovine Monocyte-Derived Macrophages (BMDM)

All experimental protocols were authorized under the UK Animals (scientific procedures) Act, 1986 and performed according to Home Office Guidelines. In addition, The Roslin Institute’s Animal Welfare and Ethics Committee (AWEC) and the AFBI Veterinary Sciences Division (VSD) Ethical Review Committee ensured compliance with all relevant legislation and promote the adoption and developments of the 3Rs. Peripheral blood from Holstein-Friesian cattle, maintained at the Roslin Institute, was collected aseptically into blood bags containing the anticoagulant CPDA-1 (Sarstedt). bovine monocyte-derived macrophages (BMDM) were generated as described previously [32] with some modifications. Briefly, peripheral blood mononuclear cells (PBMCs) were separated by density gradient centrifugation using Lymphoprep (Axis-Shield). The separated PBMCs were resuspended in serum-free RPMI-1640 (Invitrogen) at 5 × 10^6^ cells/mL and incubated for 2 h at 37 °C. The medium and non-adhered cells were replaced with RPMI-1640 supplemented with 20% fetal bovine serum (FBS), 4 mM L-glutamine, 50 mM β-mercaptoethanol, and penicillin-streptomycin (BMDM medium). The cells were cultured for 12 days at 37 °C, with a change of medium on day 7, to ensure macrophage differentiation. After 12 days, the cells were vigorously washed three times with phosphate buffered saline (PBS) to remove any non-adhered cells. The adherent cells were trypsanized by TrypLE Express (Invitrogen) and then resuspended in BMDM medium. The purity of the macrophage population was assessed using a mouse anti-bovine SIRPA (CD172a) antibody directly conjugated with RPE-Cy5 (AbD Serotec: Cat. No. MCA2041C), which confirmed that the macrophage purity exceeded 90%. All flow cytometry analyses were carried out on a CyAn flow cytometer (Beckman Coulter, Brea, CA, USA) using the Summit software. Purified monocyte-derived macrophages were resuspended at 3 × 10^5^ cells/mL in BMDM medium without antibiotics, dispensed into coverslips in 24-well plates, and cultured for 48 h before transfection.

### 2.2. Purification of Flagella

Phase one flagella were prepared from *S. typhimurium* SL1344 phase 2 mutant by shearing according to the method described by Tahoun et al. [33]. In brief, one colony of *S. typhimurium* SL1344 phase 2 mutants was stabbed in motility agar and grown overnight at 30 °C. Then, 10 μL of agar inoculums from the edge of the motility ring was cultured in LB broth overnight at 30 °C. The bacteria were harvested by centrifugation at 5000× *g* for 30 min at 4 °C. The pellet was suspended overnight at 4 °C in a volume of PBS that was 2% of the initial culture volume. The culture was sheared for 2 min on ice with an IKA T-10 homogenizer (Ultra-Turrax, Staufen, Germany) followed by centrifugation at 5000× *g* for 15 min. The supernatants were collected and re-centrifuged with the repetition of this step until obtaining a clear supernatant. The clear supernatant was centrifuged at 145,000× *g* for 90 min at 4 °C to pellet the flagella. The obtained pellet was dissolved in 500 μL PBS and stored at −20 °C until used. Flagellin protein concentration was detected by Pierce™ BCA assay kit (Thermo Scientific, Loughborough, UK) and the purity was determined by Coomassie staining of flagellin separated by SDS-PAGE.

### 2.3. Removal of LPS from Purified Flagella

Endotoxin contamination of flagella was removed according to the method described by Tahoun [33]. Briefly, 1% triton X114 was added to the purified flagella at 1 in 500. The flagella-triton mix was incubated overnight at 4 °C with rotation followed by incubation at 37 °C for 10 min and centrifugation at 11,000× *g* for 15 min. The aqueous phase at the top containing endotoxin-free flagella was collected in a new tube and the whole process was repeated a total of four times. The collected flagellin was sterilized by filtration then dialyzed to remove any remaining triton. Any remaining endotoxin was removed from the dialyzed flagellin using a column clean up Pierce^®^ High-Capacity Endotoxin binding resin (Thermo Scientific, Loughborough, UK) according to the manufacturer’s instructions. Endotoxin contamination was determined as endotoxin units (EU) per μg protein measured using an EndoLISA kit (Hyglos GmbH, Starnberger See, Germany). The values of LPS contamination in the purified flagellum were less than 0.05 EU/μg protein.

### 2.4. Lipo-Transfection of Flagella and Confocal Microscopy

The BMDM were seeded onto glass coverslips coated with mouse collagen (Sigma, Kanagawa, Japan). The cells were seeded at a density of 3 × 10^5^ per coverslip. *S. typhimurium* phase 1 endotoxin free flagellum was transfected into the macrophages at 250 ng/µL DOTAP (Roche, Basel, Switzerland). The DOTAP: flagellum mixture (1:50) was first added to FBS-free culture medium, incubated for 30 min at room temperature, and then was added to the adherent cells. The cells were washed twice with PBS and covered with 100 μL FBS-free culture medium. The macrophages were stimulated with flagellin overnight at 37 °C, 5% CO_2_, and 80% humidity. The cells were then washed three times with PBS and fixed using 4% (*w*/*v*) paraformaldehyde (pH 7.4) for 20 min, then permeabilized with 0.2% (*v*/*v*) triton X-100 in PBS for 20 min. The cells were stained with α-ASC mouse IgG (Millipore, Burlington, MA, USA; 1:100) and α-caspase 1 rabbit IgG (Santa Cruz Biotechnology, Dallas, TX, USA; 1:1000). Primary antibodies were labelled with alexa fluor 488 conjugated α-mouse IgG (Molecular Probes, Eugene, OR, USA) diluted 1:1000 and alexa fluor 594 conjugated α-rabbit IgG (Molecular Probes) diluted 1:1000. In the case of F-actin staining, the anti-ASC and anti-caspase-1 primary antibodies were labelled with alexa fluor 647-conjugated α-mouse IgG (Molecular Probes) diluted 1:1000 and alexa fluor 594 conjugated α-rabbit IgG. Then actin was stained with FITC-conjugated phalloidin diluted 1:50. Cell nuclei were stained with DAPI (Merck, Kenilworth, NJ, USA) diluted 1:5000 for 15 min, then mounted in ProLong Gold mounting medium (Invitrogen, CA, USA). To confirm the purity and identification of macrophages, the cells were stained with TRITC-conjugated anti-CD16 antibody (Santa Cruz Biotechnology). Image data was acquired with Nikon NA ×100 oil immersion lens and a multi-track (sequential scan) experimental set up on a Nikon (Tokyo, Japan), using Axiovision software.

### 2.5. Western Blot 

In order to detect the activated caspase-1, medium of the overnight flagellin-activated BMDM was collected. The flagellin transformed BMDM medium was precipitated with 10% trichloroacetic acid (TCA; Sigma) in the presence of protease inhibitor cocktail (Roche). The medium was then centrifuged at 15,000× *g* for 30 min at 4 °C. The obtained pellet was washed with 100% cold acetone and centrifuged at 15,000× *g* for 15 min at 4 °C, dried, and resuspended in 1.5M tris-HCl (pH 8.8) and the protein concentration was detected using BCA assay. The samples were denatured in a loading buffer (Sigma) and incubated at 95 °C for 5 min. The protein samples were separated by 20% SDS/PAGE, electro-transferred onto Polyvinylidene difluoride (PVDF) membranes which were blocked overnight at 4 °C in 5% nonfat dry milk. The membrane probed with polyclonal rabbit anti caspase-1 antibody (Santa Cruz Biotechnology, DallaS, TX, USA) followed by incubation with secondary mouse anti rabbit antibody. Proteins were detected by enhanced chemiluminescence.

### 2.6. Statistical Analysis

Statistical analysis of the data was performed by Student’s *t*-test using GraphPad 7 (GraphPad Software, La Jolla, CA, USA), with significance calculated at *p* < 0.05. Data are reported as mean ± standard deviation (SD). 

## 3. Results

### 3.1. Identification of BMDM

**Figure 1:**
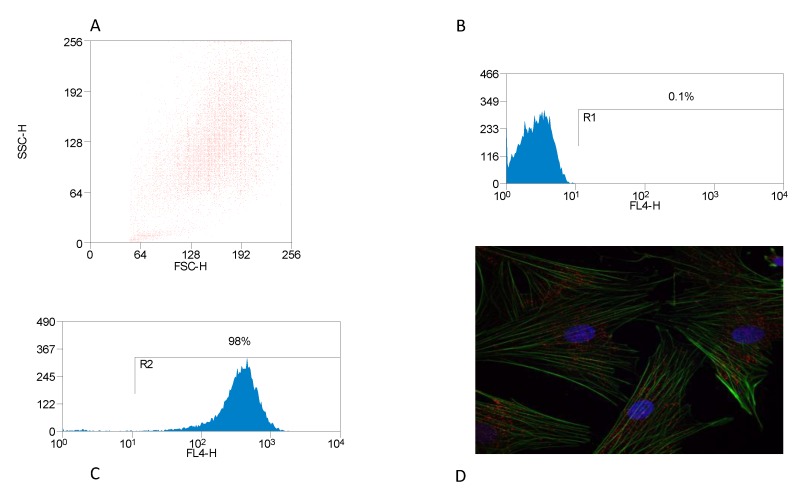
Identification of bovine macrophage. Bovine monocytes were derived from peripheral blood and differentiated into macrophages. (**A**) Forward scatter (FSC) vs. side scatter (SSC) plot of bovine monocyte-derived macrophages (BMDM), (**B**) flow cytometry analysis of unstained cells, and (**C**) flow cytometry analysis of cells stained with (SIRPA anti-bovine CD172a) antibody conjugated with RPE-Cy5. The percentage purity of the macrophage population is shown (R2). The cells were stained using TRITC-conjugated anti-CD16 antibody in combination with FITC-conjugated phalloidin for actin staining and DAPI for cell nuclei (**D**). Experiments were performed in triplicate and repeated three times (*n* = 9).

The characteristic features of BMDM cells in culture indicated that these cells are typically macrophages. The purity of the macrophage population, investigated by flow cytometry using a mouse anti-bovine SIRPA (CD172a) antibody directly conjugated with RPE-Cy5, confirmed that the macrophage purity exceeded 90% (Figure 1A–C). BMDM were phenotypically examined microscopically. BMDM were labeled with FITC-conjugated phalloidin for actin staining, TRICT-conjugated anti-CD16 antibody and DAPI for cell nuclei. The characteristic BMDM phenotypic in the staining pattern showed that these cells contain actin stress bundles and were CD16 positive (Figure 1D).

### 3.2. Inflammasome Activation and Actin Co-Localization 

**Figure 2:**
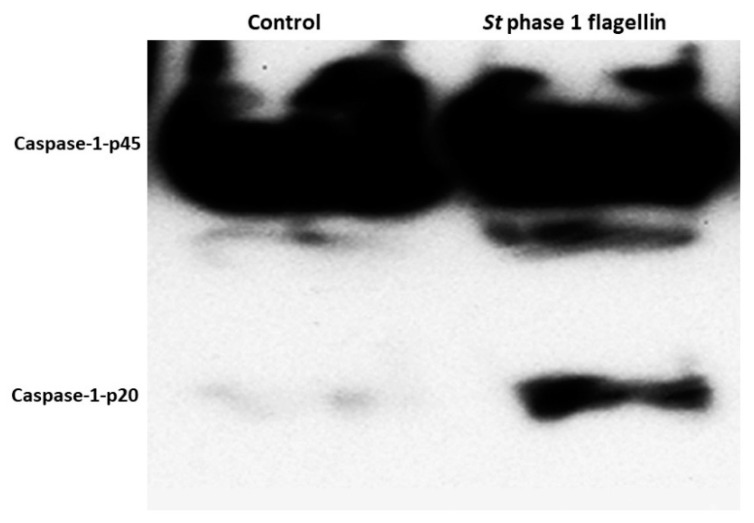
Flagellin-induced caspase-1 activation during *Salmonella typhimurium* phase 1 flagellin transfection. BMDM were stimulated with either phosphate buffered saline (PBS, control) or phase 1 flagellin overnight. The flagellin transformed BMDM medium was precipitated with 10% trichloroacetic acid (TCA), and subsequently separated using 20% SDS gels. The gels were blotted onto PVDF membranes and probed with anti-caspase-1 antibody. p20 denotes the processed mature form of caspase-1. Experiments were performed in triplicate and repeated three times (*n* = 9).

To examine whether the inflammasome was activated in BMDM by *S. typhimurium* phase one flagellin, we checked caspase-1 cleavage following exposure to flagellin. After 24 h of stimulation with flagellin, active caspase-1 detection was assessed by Western blot where the active derivative appeared with an approximate molecular weight of 20 kDa (Figure 2).

**Figure 3:**
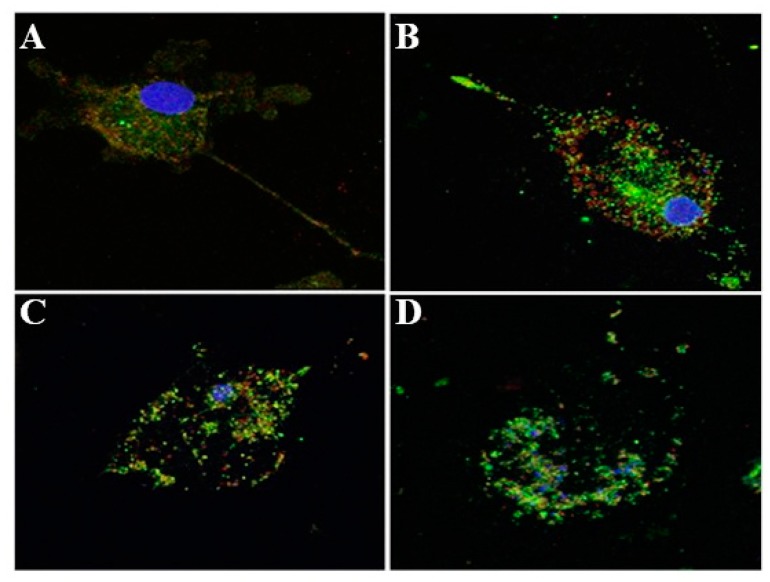

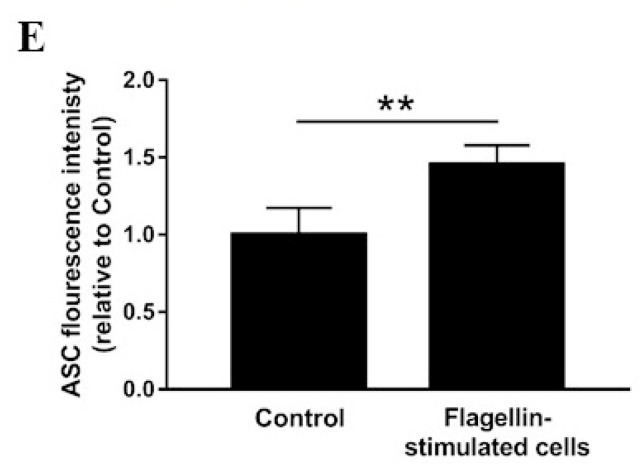
Inflammasome activation stages. BMDM were seeded on glass coverslips and challenged with *S. typhimurium* phase 1 flagellum overnight and then fixed and stained with anti-ASC (apoptosis-associated speck-like protein containing a CARD, green), anti-caspase1 (red), and DAPI nuclear stain (blue). Cells were then imaged by fluorescence confocal microscopy. Increase of ASC expression was noticed in flagellin-stimulated cells and interaction of green fluorescent ASC with the red fluorescent caspase-1 (**A**–**C**) and in the cell during the stage of pyroptosis with fragmented and scattered nuclear DNA (**D**). Quantification of ASC in flagellin-stimulated cells showed a significant increase when compared with control cells (**E**). Data are mean ± SD. ** *p* < 0.01. Experiments were performed in triplicate and repeated three times (*n* = 9).

**Figure 4:**
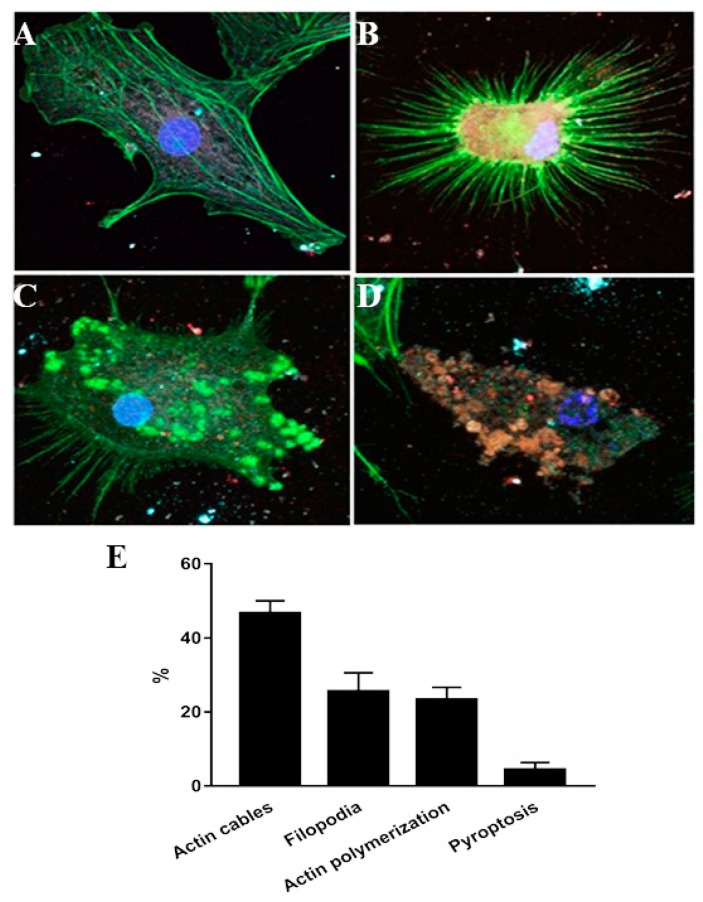
Inflammasome activation is associated with actin rearrangement. BMDM were seeded on glass coverslips and challenged with *S. typhimurium* phase 1 flagellum overnight. The cells were fixed and stained with anti ASC (cyne), anti-caspase1 (red), and DAPI nuclear stain (blue). The non-stimulated cell showed evenly distributed actin cables (**A**), whereas the flagella lipo-transformed cells showed reorganization of actin cables into filopodia (**B**), actin polymerization (**C**), and in the late stage of pyroptosis the actin cables were milted into the active inflammasome (**D**). Percentage of cells showing actin cables, filopodia, actin polymerization, and pyroptosis (**E**). Experiments were performed in triplicate and repeated three times.

To further validate these findings and to examine the stages of inflammasome activation in flagellin-challenged BMDM-derived macrophages and association of ASC with caspase-1, the BMDM cells treated with phase one flagellin for 24 h were processed for fluorescent microscopy. In these fluorescent images, green fluorescent ASC was significantly (*p* < 0.01) increased in the stimulated cells as compared to the control (Figure 3E) and was closely associated with the red fluorescent caspase-1 in the case of stimulation with flagellin (Figure 3A–C) and the cell in the stage of pyroptosis with fragmented and scattered nuclear DNA (Figure 3D). It was hypothesized that F-actin might interact with the components of active inflammasome complex that might contribute to activation of immune response. To examine this hypothesis, BMDM were treated with purified flagellin to induce inflammasome activation. The macrophages were processed for fluorescent microscopy at different stages following flagellin addition. In these fluorescent images, the inflammasome complexes alexa flour 647 stained ASC (cyan), TRITC stained caspase-1 (red) were closely associated with phalloidin-FITC-stained F-actin fibers (green) (Figure 4). The microscopic observations were also characterized by active rearrangement of actin filaments and F-actin closely associated with inflammasome complex (Figure 4B–D) while the non-stimulated cell showed evenly distributed actin cables (Figure 4A). The flagellin-challenged cells showed remolding and reorganization of actin cables into filopodia (Figure 4B,C) and in the late stage of pyroptosis the actin cables were co-localized with the active inflammasome (Figure 4D). 

## 4. Discussion

The innate immune response depends upon the ability of the host to distinguish self from non-self, and commensal organisms from pathogens to ensure that the response is appropriate [34]. Variation in these processes underlies host susceptibility to disease and different pathological outcomes from the response [35,36]. Phagocytosis represents the first line of host innate and adaptive defense against pathogens, providing a very important mean of protection for the host [37,38]. Activated macrophages degrade ingested micro-organisms by producing reactive oxygen and nitrogen metabolites [39,40,41]. Macrophages also play a role in antigen processing and presentation [42]. Most important functions depend on TLRs and inflammasomes [23,33,43]. Flagellin can activate the innate immune response via activation of inflammasomes [44,45]. Herein, we aimed to understand, at the molecular level, the functional responses of bovine macrophages to flagellin that govern the nature and level of the early innate responses against flagellated bacteria in ruminants. We have previously shown that bovine macrophages detect *Escherichia* *coli* flagellin [33], which resulted in the production of IL-8. Furthermore, we have recently shown that bovine macrophages detect flagellin from other bacteria [46], whereas the stimulation with extracellular *E. coli* flagellin did not result in the production of active IL-1β (data not shown). However, the ability of flagellin to activate inflammasomes in bovine macrophages has not been defined yet. Miao et al. [43] have previously demonstrated that cytosolic flagellin can activate inflammasomes in murine bone-marrow derived macrophages via the NLRC4 inflammasome. Here, we investigated the interaction between flagellin and inflammasomes in bovine peripheral blood monocyte-derived macrophage and showed that cytosolic flagellin can trigger inflammasome activation.

We first purified the PBMC-derived macrophages and the purity of these cells was checked by FACS using SIRPA anti-bovine CD172 antibody conjugated with RPE-Cy5. Previous studies have shown that the murine macrophages contain actin stress bundles that give the cells flexible motility which helps macrophage function. For instance, Fc-mediated phagocytosis, in which pseudopodial extensions of the cell surface cover opsonized particles and enclose them to form phagosomes, occurs by regulated actin polymerization and reorganization [47,48]. Our results represent the first evidence that BMDM also contain the actin stress bundles and this agree with macrophage functions that need activity and flexibility to deal with pathogens.

Multiple macrophage receptors, including TLRs and NLRs have been implicated in recognition of bacterial determinants resulting in cell signaling pathways that restrict the infection and activate the host immune response [8,49]. The NLRs recognition of bacterial agonists induces activation of inflammasome. The activation of inflammasome with the subsequent activation of caspase-1 and release of inflammatory cytokines have been implicated in many inflammation consequences [8,50]. Caspase-1 belongs to a family of cysteine proteases enzymes comprising single, enzymatically active p10 or p20 subunits which are responsible for the cleavage of pro-IL-1β and pro-IL-18 leading to their release [8,51]. Previous reports indicated that pro-caspase-1 interacts with ASC and NLR for inflammasome assembly and formation of the active caspase-1 [52]. The presence of cytosolic flagellin triggered pyroptotic cell death through inflammasome activation [53]. Our results indicated that activation of inflammasome resulted in cleavage of caspase-1 and the formation of active p20 subunit. Our findings also indicated that there was an increase in the expression level of ASC in flagellin-activated BMDM and the interaction between caspase-1 and ASC along with pyroptotic cell death. In the same context, Akhter et al. [54] have demonstrated that actin polymerization is induced by the interaction of inflammasomes and activated caspase-11, which led to the fusion of the *L. pneumophila* containing vacuoles with lysosomes. Our results indicated that flagellin-induced inflammasome activation induces actin rearrangement in BMDM. The actin rearrangement varied from filopodia to polymerization. Furthermore, at the stage of pyroptotic cell death, actin filaments were degraded and co-localized with activated inflammasome complex. Our results have clarified that flagellin might be endocytosed into the cytosol to activate inflammasome. These findings may be valuable in explaining how inflammation occurs in the case of infection of bovine host with flagellated bacteria such as *S. typhimurium*. Our data have indicated filopodia and interaction of actin with inflammasome in response to flagellin in pyropotitic cell death stage.

## 5. Conclusions

Our results have provided new and unequivocal evidence that actin inflammasome interaction in response to flagellin is an important part of the inflammation process. Therefore, it is noteworthy assuming that flagellin-dependent activation of the inflammasome and actin localization to the inflammasome at the stage of pyroptotic cell death are important for appropriate immune responses. These findings could be of importance for the development of preventive or therapeutic strategies for inflammatory diseases in which inflammasomes are implicated. However, further studies are needed to explore the exact mechanisms underlying the cross-linking between inflammasome complex and actin.
